# Supplementary material for: Preparation and Evaluation of Inhalable Microparticles with Improved Aerodynamic Performance and Dispersibility Using L-Leucine and Hot-Melt Extrusion
Source: Pharmaceutics. 2024 Jun 8;16(6):784. doi: 10.3390/pharmaceutics16060784 (PMC11206964; doi:10.3390/pharmaceutics16060784)
Supplement: Supplementary file 1 [file pharmaceutics-16-00784-s001.zip › pharmaceutics-3037265-supplementary.pdf]

pharmaceutics

Supplementary Materials

Preparation and Evaluation of Inhalable Microparticles with Improved Aerodynamic Performance and Dispersibility Using L-Leucine and Hot-Melt Extrusion

Jin-Hyuk Jeong [1,†], Ji-Su Kim [1,†], Yu-Rim Choi [1], Dae Hwan Shin [1], Ji-Hyun Kang [1,2], Dong-Wook Kim [3], Yun-Sang Park [4] and Chun-Woong Park [1,*]

Table S1. Formulation of ITZ microparticles according to HME temperature

Code	HME Process condition		Formulation ratio (%)		
	Tem. (℃)	rpm	Itraconazole (ITZ)	Mannitol (MAN)	L-leucine (LEU)
**HME160-L0**	**160**	**150**	20.00	80.00	**N/A**
**HME160-L0.1**			19.98	79.92	**0.10**
**HME160-L1**			19.80	79.20	**1.00**
**HME160-L10**			18.00	72.00	**10.00**
**HME200-L0**	**200**	**150**	20.00	80.00	**N/A**
**HME200-L0.1**			19.98	79.92	**0.10**
**HME200-L1**			19.80	79.20	**1.00**
**HME200-L10**			18.00	72.00	**10.00**

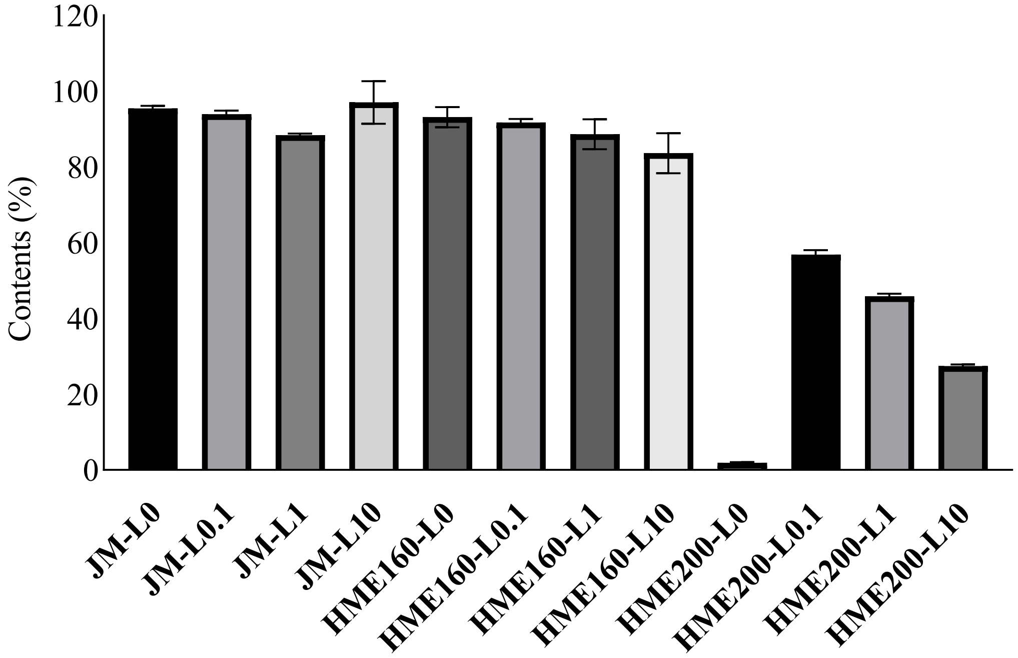

Figure S1. Contents of JMs, HME160s, HME200s

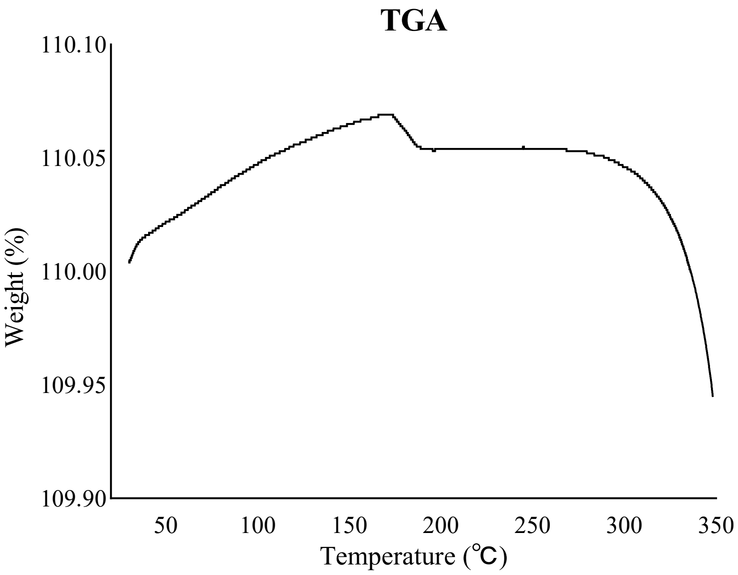

Figure S2. Thermogravimetric analysis thermogram of raw ITZ.

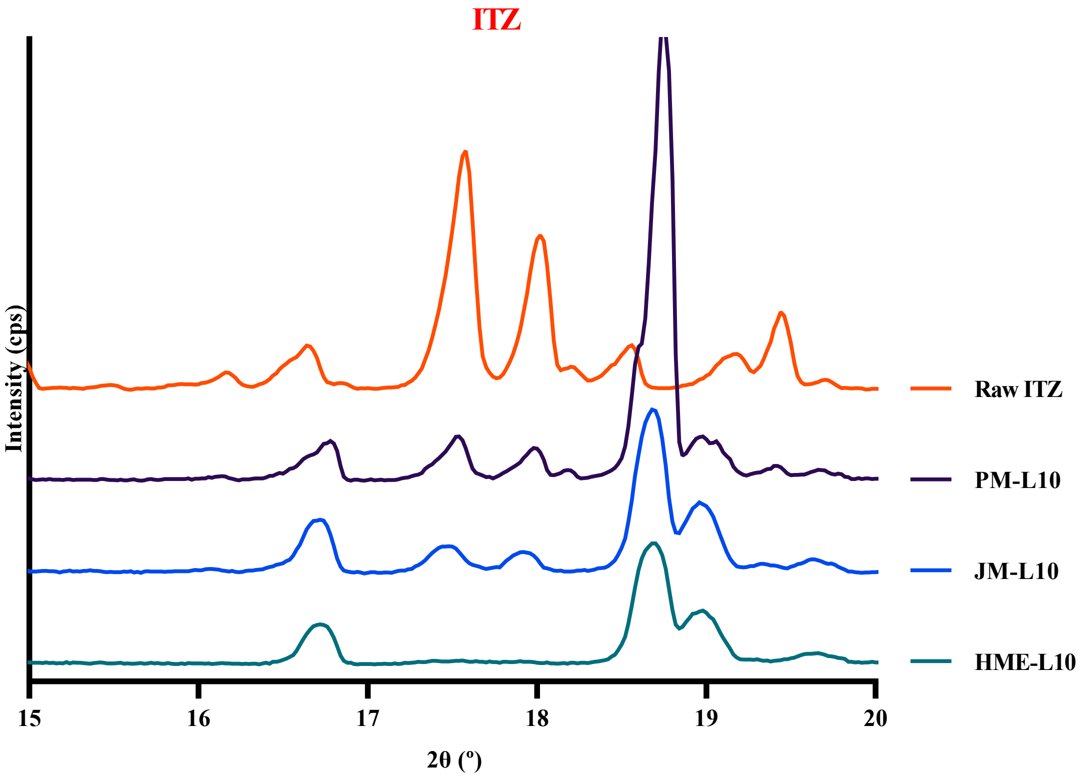

Figure S3. XRD patterns of Raw ITZ, PM-L10, JM-L10, and HME-L10 in the 15-20 º range. The red highlights indicate the characteristic peaks of ITZ.

Table S2. Flowability indicators such as Carr's index and the Hausner ratio.

Formulation	Density (g/mL)		Carr's index (%)		Hausner ratio	
	bulk	tapped				
PM-L0	0.32	0.58	45.5	Very, very poor	1.8	Very, very poor
PM-L10	0.41	0.60	32.1	Very poor	1.5	Very poor
JM-L0	0.25	0.42	40.4	Very, very poor	1.7	Very, very poor
JM-L10	0.26	0.51	48.1	Very, very poor	1.9	Very, very poor
HME-L0	0.24	0.39	39.6	Very, very poor	1.7	Very, very poor
HME-L10	0.28	0.36	22.7	Passable	1.3	Passable
